# Short-Long Heart Rate Variation Increases Dispersion of Action Potential Duration in Long QT Type 2 Transgenic Rabbit Model

**DOI:** 10.1038/s41598-019-51230-9

**Published:** 2019-10-16

**Authors:** Tae Yun Kim, Paul Jeng, JungMin Hwang, Zachary Pfeiffer, Divyang Patel, Leroy L Cooper, Konstantinos Kossidas, Jason Centracchio, Xuwen Peng, Gideon Koren, Zhilin Qu, Bum-Rak Choi

**Affiliations:** 1 Cardiovascular Research Center, Division of Cardiology, Rhode Island Hospital, Warren Alpert Medical School of Brown University, Providence, RI USA; 2 College of Pharmacy, University of Rhode Island, Kingston, RI USA; 3 Department of Cardiovascular Medicine, Heart and Vascular Institute, Cleveland Clinic Foundation, Cleveland, Ohio, USA; 4 Biology Department, Vassar College, Poughkeepsie, NY USA; 5 Department of Comparative Medicine, Pennsylvania State University College of Medicine, Hershey, PA USA; 6 Department of Medicine (Cardiology), David Geffen School of Medicine, University of California, Los Angeles, CA USA

**Keywords:** Arrhythmias, Cardiovascular diseases

## Abstract

The initiation of polymorphic ventricular tachycardia in long QT syndrome type 2 (LQT2) has been associated with a characteristic ECG pattern of short-long RR intervals. We hypothesize that this characteristic pattern increases APD dispersion in LQT2, thereby promoting arrhythmia. We investigated APD dispersion and its dependence on two previous cycle lengths (CLs) in transgenic rabbit models of LQT2, LQT1, and their littermate controls (LMC) using random stimulation protocols. The results show that the short-long RR pattern was associated with a larger APD dispersion in LQT2 but not in LQT1 rabbits. The multivariate analyses of APD as a function of two previous CLs (APD_n_ = C + α_1_CL_n−1_ + α_2_CL_n−2_) showed that α_1_ (APD restitution slope) is largest and heterogeneous in LQT2 but uniform in LQT1, enhancing APD dispersion under long CL_n−1_ in LQT2. The α_2_ (short-term memory) was negative in LQT2 while positive in LQT1, and the spatial pattern of α_1_ was inversely correlated to α_2_ in LQT2, which explains why a short-long combination causes a larger APD dispersion in LQT2 but not in LQT1 rabbits. In conclusion, short-long RR pattern increased APD dispersion only in LQT2 rabbits through heterogeneous APD restitution and the short-term memory, underscoring the genotype-specific triggering of arrhythmias in LQT syndrome.

## Introduction

Long-QT syndrome (LQTS) is an inherited disease associated with prolongation of QT interval and sudden cardiac death (SCD)^1^. The two most common forms of LQTS are caused by mutations in the KCNQ1 (LQT1) and KCNH2 genes (LQT2), which encode the α subunits of the slowly activating (I_Ks_) and rapidly activating (I_Kr_) voltage-gated potassium channels, respectively. Clinical studies have documented that pause-dependent initiation of polymorphic ventricular tachycardia (pVTs) is associated with either congenital LQT2^2,3^ or acquired LQT2^4^, and is classically referred to as the short-long-short RR interval sequence of R-on-T early afterdepolarization (EAD)^2,3,5^.

The ‘pause’, or short-long initiation pattern, can cause excessive APD prolongation in LQTS to promote EADs, leading to pVTs. In addition, ‘pause’ may increase APD dispersion, a substrate vulnerable to reentry by causing conduction blocks in LQT syndromes^6,7^. Indeed, several groups have shown that a pause can increase APD dispersion in drug-induced experimental animal models of LQTS^7,8^. In addition to well-recognized role of APD dispersion in reentry formation, our group also reported that the enhanced APD dispersion could promote the spontaneous genesis of premature ventricular complexes (PVCs) by electrotonic current flow in the steep repolarization gradient area^9,10^, suggesting that APD dispersion can act as both substrate and trigger for pVT initiation in LQTS.

Despite the importance of APD dispersion in LQT-related arrhythmias, it is not clear whether the short-long cycle length (CL) pattern increases APD dispersion as well as how varied CL can effect on behavior of APD dispersion. To understand how APD dispersion is modulated under alternating CL pattern, the effect of previous multiple CL changes on APDs should be considered. A typical APD restitution curve as a function of a single diastolic interval from a S1S2 protocol is significantly limited in this case, since it does not account for the effect of short-long alternating CL. Previous studies using two premature stimuli (S1-S2-S3 intervals) showed that the APD dynamics of S3 is markedly different from the APD restitution curve of the S2 beat^11–13^.

Several groups have introduced the term ‘short-term cardiac memory’ which refers to the effects of pacing history on APD^14–18^. Theoretical and experimental studies used random CL pacing^19^ or stochastic pacing protocol^20–22^ to investigate APD modulation by pacing history and its influence on alternans. The adaptation of APD to history of CL change can be attributed to the activation/inactivation and recovery kinetics of ionic currents and Ca^2+^ handling, and changes in intracellular and extracellular ion concentration. Ion channel kinetics most likely underlies short-term cardiac memory and it is possible that prior heart rate variations such as short-long CL may greatly influence APD dynamics in LQTS lacking I_Ks_ or I_K r_ and is probably an important factor in determining APD dispersion under characteristic short-long RR intervals as seen in LQT2 but not in LQT1 patients. However, most of studies on short-term memory focused on modulation of APD dynamics and alternans by pacing history and its effect on APD dispersion in LQTS in a genotype-specific manner is not fully elucidated.

Telemetry recordings from LQT2 rabbits showed characteristic short-long CL variation before the onset of pVT and sudden cardiac death^23,24^ similar to LQT2 patients^2,3^. We therefore used these transgenic rabbits to investigate the mechanisms underlying CL–dependent APD dispersion in LQTS. APD dispersion dynamics from littermate control (LMC), LQT1 and LQT2 rabbits were studied using randomly varying CLs and short-long CL pacing protocol and we found that APD dispersion in LQT2 rabbits is highly dependent on a previous history of heart rate variation due to heterogeneous restitution and short-term cardiac memory.

## Results

### CL patterns preceding maximum APD dispersion

**Figure 1:**
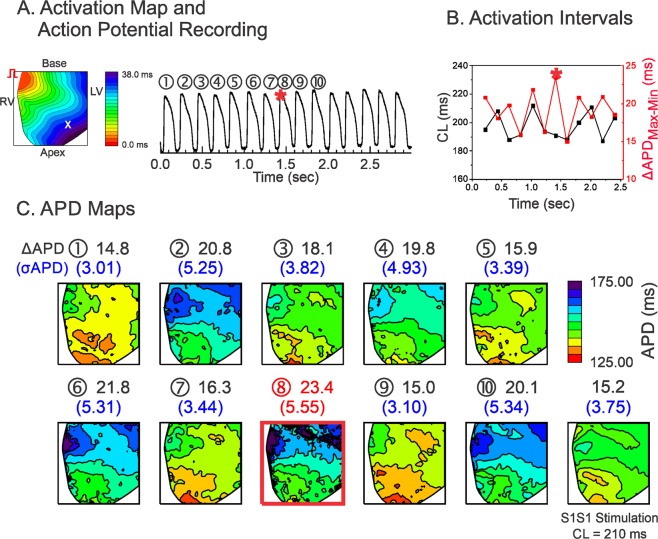
Influence of CL variation on APD dispersion in LMC. (**A**) Activation map and sample trace of action potentials during random stimulation. (**B**) Corresponding mean CLs (black) and ΔAPD_max-min_ (red) in panel (A,C) APD maps corresponding to the action potentials shown in panel A. ΔAPD_max-min_ and the standard deviations of the APDs (σ_APD_) are shown above the corresponding maps. The beat with maximum APD dispersion is marked with a red square (the 8^th^ beat, ΔAPD_max-min_ = 23.4 ms). The corresponding beat of the maximum ΔAPD_max-min_ is marked with a red star in panels A and B. The APD map from S1S1 stimulation at the closest average CL of random stimulation protocol is also shown at the end for comparison (CL = 210 ms in this scan, ΔAPD_max-min_ = 15.2 ms, σ_APD_ = 3.75 ms).

Hearts of all three genotypes (LMC, LQT1, and LQT2) were stimulated using a series of computer-generated random CLs in the range of tissue refractoriness plus 50 ms to investigate dynamics of APD dispersion under various CL combinations (see Methods). Figure 1 shows typical examples of activation map, APD traces, CL variation, and APD maps from a LMC rabbit. Figure 1A shows an activation map and an AP trace from one mapping site (x), and Fig. 1B shows the CLs measured from the same site. Figure 1C shows the series of APD maps and APD dispersion for 10 consecutive beats. In this case, the maximum APD dispersion occurred at the 8^th^ beat (marked with a red star in panels A–C). The last panel in Fig. 1C is the APD map from constant rate pacing at CL = 210 ms (=averaged CL of random pacing).

**Figure 2:**
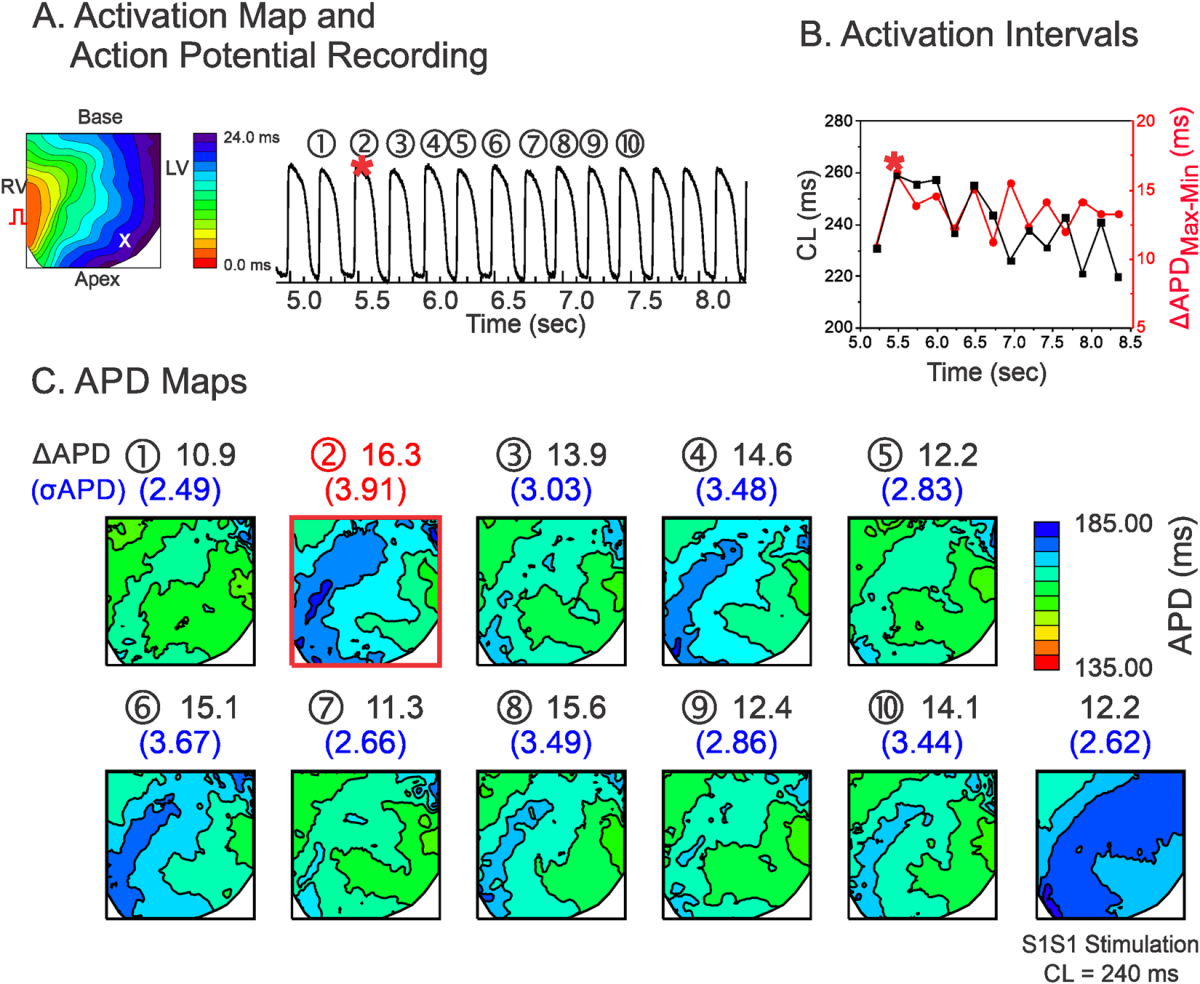
Influence of CL variation on APD dispersion in LQT1. (**A**) Activation map and sample trace of action potentials. (**B**) Corresponding mean CLs (black) and ΔAPD_max-min_ (red) in panel (A,C) APD maps for each stimulation in A. The beat with maximum APD dispersion are marked with a red square (2^nd^ beat, ΔAPD_max-min_ = 16.3 ms, σ_APD_ = 3.91 ms). Prior to the maximum APD dispersion, CLs did not show a short-long CL pattern.

**Figure 3:**
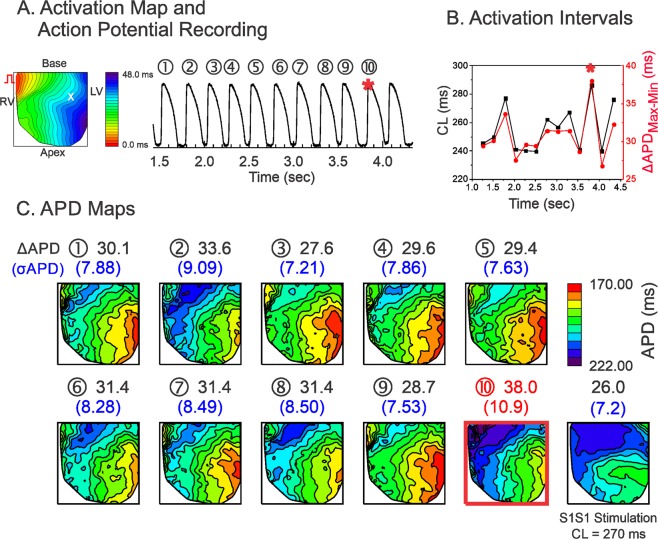
Influence of CL variation on APD dispersion in LQT2. (**A**) Activation map and sample trace of action potentials. (**B**) Corresponding mean CLs (black) and ΔAPD_max-min_ (red) in panel (A,C) APD maps. The beat with the maximum APD dispersion is marked with a red square (10^th^ beat). Note that short-long alternating CL precedes the maximum APD dispersion.

Figure 2 shows a representative result from a LQT1 heart. APD dispersion in the LQT1 hearts was smaller overall than that in the LMC hearts. Long CLs repeatedly produced higher APD dispersion in LQT1. Figure 3 shows an example from a LQT2 heart. The maximum dispersion in LQT2 (σ_APD_ = 10.9 ms, ΔAPD_max-min_ = 38.0 ms) occurred at the 10^th^ beat. The dispersion under ramp pacing (panel C, last APD map at CL = 270 ms, σ_APD_ = 7.2 ms, ΔAPD_max-min_ = 26.0 ms) was smaller than that under random pacing, suggesting that CL variations were more likely to promote APD dispersion as compared to fixed CL.

Overall, the APD dispersion is largest in LQT2 and smallest in LQT1. The beat-to-beat APD variation was greatest in LQT2, medium in LMC, and lowest in LQT1 hearts (see Supplementary Fig. S1 for expanded traces and Table 1 for summary of APD dispersion from three genotypes).

**Table 1:** Summary of statistical data analysis.

	LMC	LQT1	LQT2
Max σ_APD_ (Figs 1–3)	Fig. 1. Under Random Stimulation: σ_APD_ = 5.55 ms ΔAPD_max-min_ = 23.4 ms Under S1S1 Stimulation: CL = 210 ms σ_APD_ = 3.75 ms ΔAPD_max-min_ = 15.2 ms	Fig. 2. Under Random Stimulation: σ_APD_ = 3.91 ms ΔAPD_max-min_ = 12.2 ms Under S1S1 Stimulation: CL = 240 ms σ_APD_ = 2.62 ms ΔAPD_max-min_ = 12.2 ms	Fig. 3. Under Random Stimulation: σ_APD_ = 10.9 ms ΔAPD_max-min_ = 38.0 ms Under S1S1 Stimulation: CL = 270 ms σ_APD_ = 7.2 ms ΔAPD_max-min_ = 26.0 ms
Beat-to-beat APD variation (standard deviation of APD during random stimulation,σ_beat-to-beat_) (Figs 1–3)	5.8 ± 1.2 ms n = 5 hearts in Fig. 1	3.2 ± 0.9 ms n = 5 hearts in Fig. 2	8.8 ± 2.0 ms n = 5 hearts in Figs 1–3 LMC vs. LQT2, p < 0.05 LQT1 vs. LQT2, p < 0.05 (Student’s t-test respectively)
ΔCL = CL_n−1_ – CL_n−2_ at maximum APD dispersion (paired t-test between CL_n−1_ and CL_n−2_) (Fig. 4A)	5.3 ± 17.7 ms *p* = 0.2084 n = 5 hearts, 10 scans	5.0 ± 18.6 ms *p* = 0.2084 n = 5 hearts, 10 scans	17.7 ± 14.1 ms CL_n−1_ > CL_n−2,_ p < 0.01 n = 5 hearts, 10 scans
Correlation of σAPD vs. ΔCL under random stimulation (Fig. 4B)	0.008 ± 0.002 r = 0.23 ± 0.14 n = 4 hearts LMC vs. LQT1, p < 0.05	0.002 ± 0.004 r = 0.10 ± 0.16 n = 4 hearts	0.022 ± 0.008 r = 0.51 ± 0.17 n = 4 hearts LMC vs. LQT2, p < 0.05 LQT1 vs. LQT2, p < 0.05 (Student’s t-test respectively)
Correlation between ΔCL and σ_APD_ under S1S2S3 stimulation (Fig. 4C)	r = 0.68 ± 0.09 *p* < 0.05 n = 4 hearts	N/A	r = 0.79 ± 0.14 *p* < 0.01 n = 4 hearts
Slope of σ_APD_ vs. ΔCL under S1S2S3 stimulation (Fig. 4C)	0.005 ± 0.001 n = 4 hearts	N/A	0.035 ± 0.020 LMC vs. LQT2, *p* *<* 0.01 n = 4 hearts (Student’s t-test)
Coefficient α_1_ under random stimulation in Fig. 5C (Statistical differences were found by one-way ANOVA test at p = 0.05 level)	0.23 ± 0.06 n = 6 hearts	0.17 ± 0.02 n = 6 hearts LMC vs LQT1, p = 0.029 (Student’s t-test)	0.32 ± 0.06 n = 6 hearts LMC vs LQT2, p = 0.039 LQT1 vs LQT2, p < 0.00003 (Student’s t-test respectively)
Coefficient α_2_ under random stimulation in Fig. 5C (Statistical differences were found by one-way ANOVA test at p = 0.05 level)	−0.068 ± 0.04505 n = 6 hearts	0.007 ± 0.051 n = 6 hearts LMC vs LQT1, p = 0.023 (Student’s t-test)	−0.053 ± 0.021 n = 6 hearts LQT1 vs LQT2, p = 0.025 LMC vs LQT2, p = 0.461 (Student’s t-test)
Standard deviation of restitution slope map under random stimulation in Fig. 5D	0.030 ± 0.014 n = 4 hearts	0.029 ± 0.008 n = 4 hearts	0. 064 ± 0.025 n = 4 hearts LMC vs. LQT2, *p* < 0.05 (Student’s t-test)
Correlation between α_1_ and α_2_ in Fig. 5E	−0.07 ± 0.15 n = 6 hearts	N/A	−0.27 ± 0.10 n = 6 hearts

### Short-long alternating CL increases APD dispersion only in LQT2 hearts

**Figure 4:**
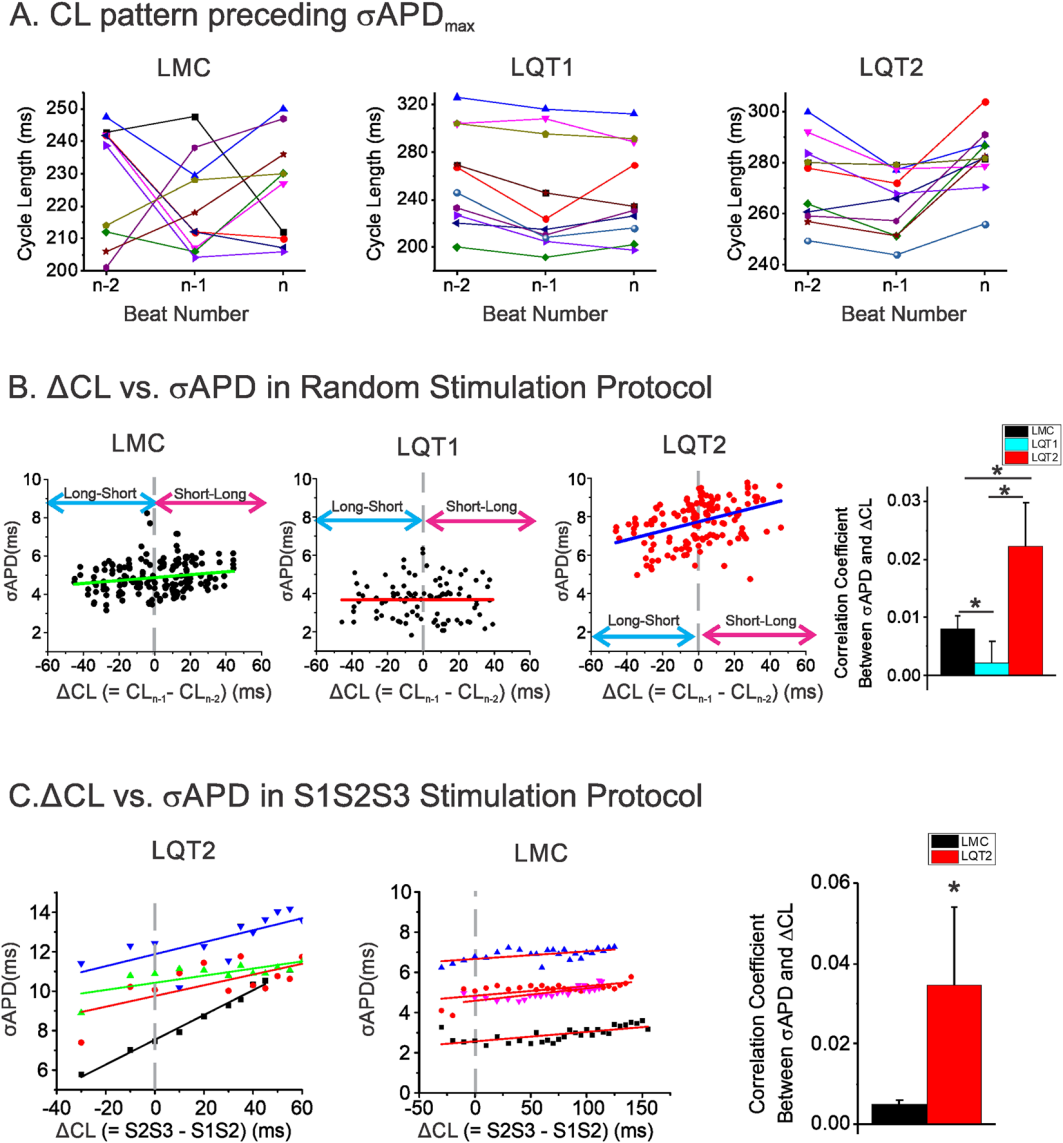
CL patterns preceding the maximum APD dispersion. (**A**) Three previous CLs (CL_n−3_ − CL_n−2_ − CL_n−1_) preceding ΔAPD_max_ in LMC, LQT1, and LQT2 (n = 10 stimulation protocols from 5 hearts per group). Note that long-short-long CL patterns precede ΔAPD_max_ in LQT2 (paired t-test between CL_n−2_ and CL_n−1_, p = 0.0017), while no clear pattern was found in LMC (p = 0.1836) or LQT1 (p = 0.2084). (**B**) Correlation analysis of alternating CL and APD dispersion in random stimulation protocol. CL difference (ΔCL = CL_n−1_ − CL_n−2_) vs. APD dispersion (σ_APD_) shows a clear positive association in LQT2, unlike LMC & LQT1. Correlation coefficient between APD dispersion (σ_APD_) and ΔCL in LQT2 greater than others under random stimulations (slope = 0.00797 ± 0.00237 in LMC, 0.0022 ± 0.0037 in LQT1, and 0.02218 ± 0.00759 in LQT2 n = 4 hearts each. p = 0.039, 0.012, and 0.003 in LMC VS. LQT1, LMC VS LQT2 and LQT1 VS. LQT2 respectively, Student’s t-test where appropriate). APD dispersion of LQT2 is more dependent on CL difference. (**C**) Correlation of CL difference (ΔCL = S2S3 − S1S2) with APD dispersion (σ_APD_) in S1S2S3 Protocol replicating short-long CL pattern. In LQT2, there is a positive relationship (slope = 0.035 ± 0.020, n = 4 hearts) between short-long cycle and APD dispersion (σ_APD_), but of much smaller size in LMC (slope = 0.003 ± 0.003, n = 4 hearts, p = 0.02 LQT2 VS. LMC, Student’s t-test where appropriate).

Figure 4A shows CLs from the three previous CLs preceding the beat exhibiting maximum APD dispersion from LMC, LQT1, and LQT2 hearts. LQT2 hearts exhibited a long-short-long CL sequence (short-long if only two previous CLs included) that preceded the beat with maximum APD dispersion, while LMC and LQT1 hearts did not show clear CL dependent patterns (Table 1). To further quantify the effect of alternating CL on APD dispersion, we investigated whether there is any correlation between CL difference (ΔCL = CL_n−1_ − CL_n−2_, a degree of CL change from short to long) and APD dispersion. Panel B shows typical examples of APD dispersion (σ_APD_, see Methods) vs. ΔCL from 20-second scans in LMC, LQT1, and LQT2 hearts. The results show a positive correlation between ΔCL and APD dispersion in LQT2 but negligible correlation in LMC and LQT1 (Table 1). APD dispersion in LQT2 is more dependent on a degree of CL change.

To further verify that a short-long CL increases APD dispersion in LQT2, we applied an S1S2S3 pacing protocol in which S2S3 was fixed while S1S2 was variable (see Methods). This stimulation protocol evaluates the effect of S1S2 on APD dispersion of the S3 beat, since S2S3 is fixed. Figure 4C shows the results of APD dispersion of the S3 beat from LMC and LQT2 (n = 4 per group). To be consistent with panel B, we used ΔCL = S2S3 − S1S2 for the *x*-axis, and S2S3 was fixed at 320 ms. Both LQT2 and LMC demonstrated statistically significant correlation between ΔCL and σ_APD_ (Table 1). The linear regression of σ_APD_ vs. ΔCL showed a steeper slope in LQT2 (Table 1). Thus, shortening S1S2 by 100 ms increased APD dispersion by 19 ms in LQT2 but only 2.7 ms in LMC. These results strongly suggest that S1S2 alone has a significant influence on APD dispersion of the S3 in LQT2 rabbits, setting the stage for the generation of pVTs.

### Genotypic specific restitution kinetics of APD and short-term memory

**Figure 5:**
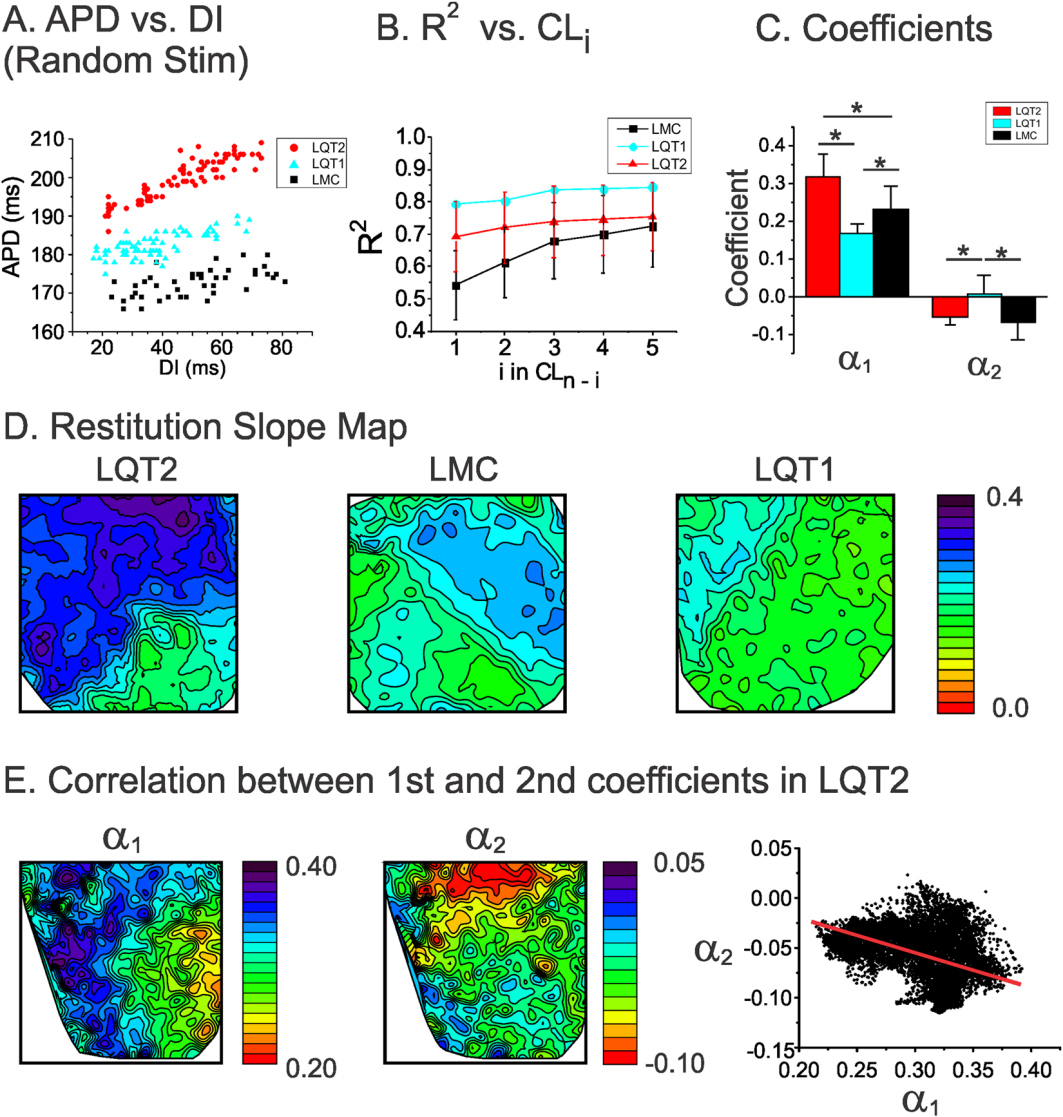
APD restitution and short-term memory effect on APD dispersion. (**A**) Examples of APD restitution from LMC, LQT1, and LQT2 hearts using random stimulation protocol. Note that the restitution slope is greatest in LQT2 followed by LMC and LQT1. (**B**) Goodness of fit (R^2^) of multivariate analysis using $AP{D}_{n}=\,C+{\alpha }_{1}C{L}_{n-1}+{\alpha }_{2}C{L}_{n-2}+\,\cdots +\,{\alpha }_{k}C{L}_{n-k}\,\,$where *k* is the k^th^ previous beats. R^2^ including previous 10 beats were 0.848 ± 0.043, 0.85 ± 0.002, and 0.792 ± 0.093 for LMC, LQT1, and LQT2 respectively. (**C**) Coefficients (α_1_ & α_2_) from multivariate regression analysis (* indicates p < 0.05, One-Way ANOVA tests were performed between three groups and significant difference were founded at the 0.05 level. The differences of α_1_ in LMC vs LQT1, LQT1 vs LQT2 and LMC vs LQT2 are significant at the 0.05 level by Fisher test and the differences of α_2_ in LMC vs LQT1 and LQT1 vs LQT2 are significant at the 0.05 level by Fisher test). (**D**) Spatial patterns of 1^st^ coefficients (α_1_) in LMC, LQT1, and LQT2 hearts. Note that the map of α_1_ from LQT2 shows greater spatial heterogeneities (0.09–0.39) compared to that of LMC and LQT1 hearts (standard deviation of slope map = 0. 064 ± 0.025 in LQT2 vs. 0.029 ± 0.008 and 0.030 ± 0.014 in LQT1 and LMC, n = 4 hearts each, p < 0.05, Student’s t-test where appropriate). (**E**) Spatial patterns of 1^st^ and 2^nd^ coefficients (α_1_ & α_2_) in LQT2. The map of α_2_ shows a spatial gradient with higher slope at the apex than the base, different from the α_2_ map. The α_1_ vs. α_2_ plot in the right panel shows negative correlation (−0.27 ± 0.10 in LQT2 vs. −0.07 ± 0.15 in LQT1, *p* < 0.05, n = 6 hearts, Student’s t-test where appropriate), indicating that the gradient direction in α_2_ is opposite that in α_1_.

Since the previous two CLs have greater impact on APD dispersion in LQT2 hearts, we hypothesized that short-term cardiac memory is greater in LQT2 hearts to cause large APD dispersion under the characteristic short-long CL pattern. We investigated the short-term memory effect, specifically how much the second previous CL (CL_n−2_) influences APD, using multivariate regression analysis (see Methods). Figure 5A shows typical examples of APD vs. diastolic interval (DI) scatter plots using the random pacing protocol in LMC, LQT1, and LQT2 hearts. The goodness of fit (R^2^) increases with increasing the number of previous CLs included in the multivariate analysis (panel B). The coefficients from the multivariate regression analysis are shown in panel C. The results show that the coefficient α_1_ for the first CL was greatest in LQT2, meaning that APD restitution is steepest in LQT2. The coefficient α_2_ (representing the short-term memory effect) for the second CL of LMC and LQT2 were negative (details are in Table 1) while the coefficient for LQT1 is close to zero (Table 1), meaning that shorter CL_n−2_ is associated with longer APD_n_ in LMC and LQT2.

### Heterogeneous restitution and short-term memory increases APD dispersion under short-long CL combination in LQT2

We previously reported that LQT2 hearts exhibited heterogeneous APD restitution associated with increased vulnerability to discordant alternans and reentry formation^25^. Heterogeneous restitution was also observed during the random stimulation protocol, manifesting as steeper APD restitution slope at the base than that at the apex in LQT2 (left panel in Fig. 5D). In contrast, LMC and LQT1 show much less slope dispersion (middle and right maps in panel D), indicating that APD restitution is more heterogeneous in LQT2.

We further examined whether the short-term cardiac memory effect was heterogeneous in LQT2. Figure 5E shows the α_1_ and α_2_ maps from multivariate analysis (see Methods). The α_2_ map (middle) shows heterogeneity between apex and base, indicating that the short-term cardiac memory effect in LQT2 is also heterogeneous. Interestingly, the gradient pattern of the α_2_ map (middle) is not the same as that of the α_1_ map (left). The correlation between α_1_ and α_2_ is negative, indicating that the region of steep APD restitution has a larger short-term memory effect that can dampen the APD prolongation caused by a pause.

The gradient pattern of APD dispersion is determined mainly by APD restitution slope of the first beat (α_1_). Since α_2_ inversely correlates with α_1,_ α_2_ (short-term memory) negatively impact on APD, dampening APD dispersion in LQT2. When the 2^nd^ previous CL is short, this dampening effect is abolished, leaving full exposure of APD dispersion in LQT2 rabbits (see Fig. 7 and discussion for detail).

**Figure 6:**
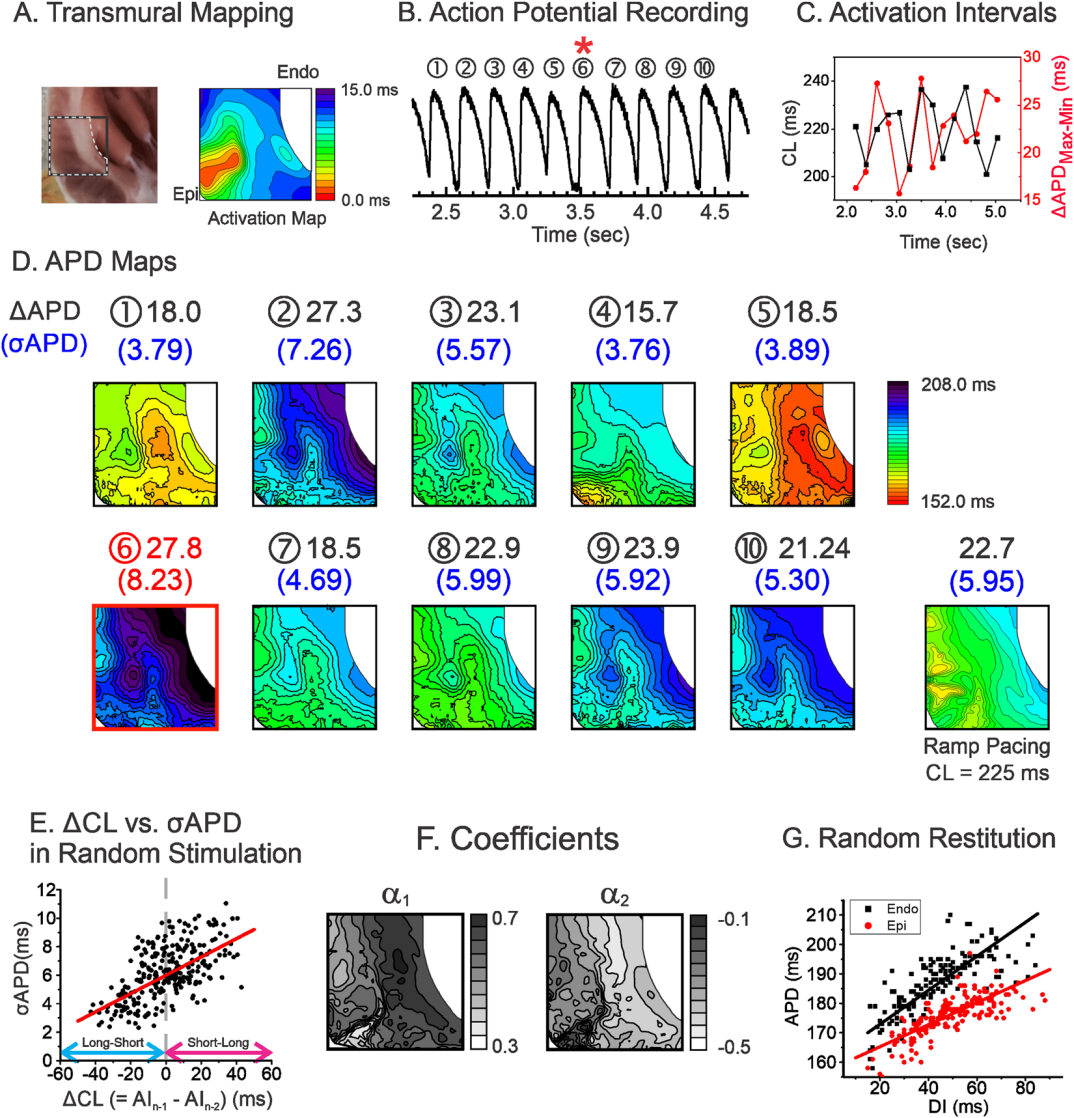
Influence of CL variation on APD dispersion across epicardium and endocardium of LV wedge preparation of LQT2 heart. (**A**) Location of transmural mapping and a typical example of activation map paced from the epicardium. (**B**) Trace of action potentials during random stimulation. (**C**) Corresponding mean CLs (black) and ΔAPD_max-min_ (red) in panel (B,D) APD maps corresponding to the action potentials shown in panel B. The beat with maximum APD dispersion is marked with a red square (the 6^th^ beat, ΔAPD_max-min_ = 27.8 ms, σ_APD_ = 8.23 ms). The corresponding beat of the maximum APD dispersion is marked with a red star in panels B and C. The APD map from S1S1 pacing at a CL closest to the average CLs of random stimulation protocol is shown in the last panel (CL = 225 ms in this scan, ΔAPD_max-min_ = 22.7 ms, σ_APD_ = 5.95 ms). (**E**) Correlation analysis of alternating CL preceding σ_APD_ in random stimulation protocol. ΔCL vs. σ_APD_ shows a positive association similar to the result from the anterior region of LQT2 in Fig. 3. (**F**) Spatial patterns of 1^st^ and 2^nd^ coefficients (α_1_ & α_2_) in the transmural region of LQT2. (**G**) Scatter plot of APD vs. DI from the base and apex showing heterogeneous restitution in LQT2. The random restitution slope of the endocardium (0.58) is steeper than that of the epicardium (0.37).

We further investigated APD dispersion dynamics under CL variations across the endocardium to the epicardium in the left ventricles (LV) of LQT2 hearts (n = 3). Figure 6 shows an example of APD dispersion recorded from LV wedge preparation during the random pacing protocol. The maximum APD dispersion of LV wedge preparation was also associated with a short-long CL pattern (Fig. 6C). The multivariate analysis revealed that the coefficient α_1_ was 0.502 ± 0.130 and α_2_ was negative (−0.213 ± 0.100, Fig. 6F). The heterogeneities of α_1_ and α_2_ were also present transmurally; see Fig. 6G, endocardium = 0.578 (black) vs. epicardium (red) = 0.375. The spatial correlation between α_1_ and α_2_ was negative (−0.847 ± 0.067), indicating that the region with a steeper APD restitution (endocardium) has a larger short-term cardiac memory effect than the region with a shallower APD restitution (epicardium). The combined epicardial and transmural mappings show that a short CL_n−2_ weakens the short-term memory effect that decreases APD dispersion caused by large APD restitution heterogeneity in LQT2.

## Discussion

APD dispersion and steep restitution have long been suspected as mechanisms underlying long QT-related arrhythmias in clinical, drug/transgenic animal and computation modeling studies^7,9,10,23,26–30^. We investigated rate-dependent APD dispersion dynamics in transgenic rabbit models of LQTS using a comprehensive stimulation protocol and found that LQT2 rabbits (but not LQT1 rabbits) show enhanced APD dispersion in response to short-long CL changes due to heterogeneous APD restitution and the short-term cardiac memory effect.

Different types of LQTS may have different mechanisms of initiation and maintenance of arrhythmias^31,32^. Sympathetic tone such as exercise is a predominant trigger in LQT1, while majority of cardiac events in LQT2 patients occur during rest/sleep or sympathetic surge such as an auditory stimulus^32^. The majority of documented arrhythmias in congenital long QT syndrome patients were ‘pause’ dependent. LQT2 patients show short-long-short initiation pattern preceding pVTs, while increasing CLs are found in LQT1 patients^2,3^, associate with differential sympathetic trigger conditions. We previously reported that the short-long pattern preceded pVT initiation in LQT2 but not in LQT1 rabbits^23,24^, suggesting that ‘pause’ is a unique feature of pVT initiation in LQT2.

Computer modeling and experimental data^7,33–35^ suggest that the post-pause prolongation of APD is a major reason for EAD formation through providing time for the recovery and the reactivation of L-type Ca^2+^ current, which generates depolarizing currents of EADs. In addition to the effect of the immediate pause on APD, our multivariate analysis of APD restitution shows the importance of two previous CLs, short-long CLs, that prolong APD even more. The prolongation of APD by short-long CLs may further increase the risk for EAD formation in LQT2.

The hallmark of substrates for arrhythmias is dispersion of repolarization, which allows unidirectional conduction block and reentry formation^36–40^. In addition, large APD dispersion can initiate tissue-scale PVCs^9,10^, suggesting large APD dispersion itself can act as both reentrant substrate and trigger. Indeed, dispersion of repolarization has been implicated as an underlying mechanism of LQT-related arrhythmias in both previous pharmacological and transgenic animal models^6,23,27,41–43^. *I*_*kr*_ blockade using E-4031 or sotalol increases the transmural APD gradient in canine hearts^8,44^ and between the apex and base in rabbit hearts^41^ and LQT transgenic mice^27^.

APD dispersion in LQTS can be dynamic, and several groups have shown a close correlation between ‘pause’ and greater APD dispersion in drug-induced animal models of LQTS^7,8^. The current study investigates the dynamic modulation of APD dispersion in the heart with varying heart rates. In addition to a simple ‘pause’-dependent increase of APD dispersion, we found that short CL before the pause can further enhance APD dispersion in LQT2. Compared to LQT1, APD and its dispersion in LQT2 are largely dependent on multiple previous CLs (Fig. 4A–C). Although LQT1 had longer APD than LMC in our transgenic models, LQT1 did not show short-long CL-dependent APD dispersion, in line with clinical observations that the short-long pattern was limited to LQT2 patients^2,3^.

The short-term cardiac memory effect can be considered an additional factor enhancing APD dispersion in LQT2 as well as heterogeneous APD restitution (Fig. 5D). Our experimental results show that APDs are dependent on at least two previous CLs (Fig. 5A–C). The coefficients from multivariate regression analysis show that 1) two previous CLs are enough to account for most of APD variation (R^2^ > 0.7); 2) LMC and LQT2, but not LQT1, are inversely influenced by the second previous CLs (α_2_ < 0); and 3) the α_2_ map does not resemble the α_1_ map, suggesting a heterogeneous short-term memory effect.

**Figure 7:**
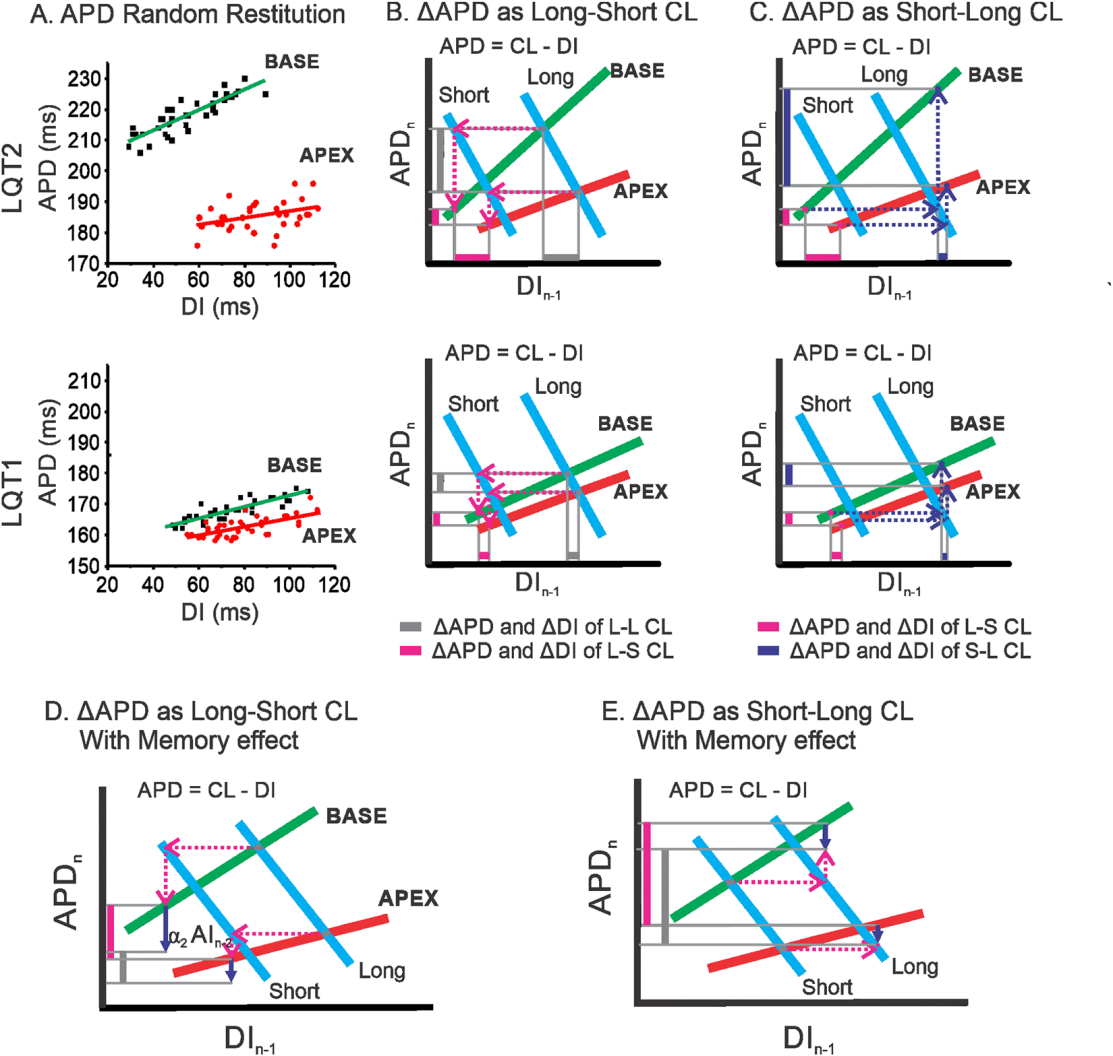
Mechanisms of short-long CL enhancing APD dispersion in LQT2 (*top*) but not in LQT1 (*bottom*). (**A**) Scatter plot of APD vs. DI from base and apex showing heterogeneous restitution in LQT2, while heterogeneity in restitution is small in LQT1. (**B**) Illustration of enhanced APD dispersion under long-short CL. The red/green lines represent apex and base APD restitution curves, and the blue lines are the plots of the functional relation between APD and DI for a fixed CL, i.e., APD = CL − DI. The vertical bars indicate APD dispersion from long-long (grey) followed by a single short CL (magenta). (**C**) Enhanced APD dispersion in LQT2 under short-long CL changes due to heterogeneous restitution (purple vertical bar). (**D**) Illustration of APD dispersion reduced by the short-term memory effect in short-long CL. (**E**) Illustration of APD dispersion reduced by the short-term memory effect in long-short CL. The purple downward arrows indicate the APD adaptation direction and magnitude by the short-term memory effect of the previous CL (${\alpha }_{1}C{L}_{n-2}$). In short-long CL, APD dispersion is less reduced by the short-term memory effect than in long-short CL. The step-by-step animation for this figure is available as a supplementary movie.

Figure 7 illustrates how heterogeneities in APD restitution and short-term memory develop greater APD dispersion in LQT2 by short-long RR intervals but not in LQT1 (the step-by-step progress of the iterations from basal CL to short and long CL changes is available as an online supplementary movie). Two restitution curves are shown in Fig. 7A, representing heterogeneous APD restitution from base (green) and apex (red) in LQT2 (top) and LQT1 (bottom). LQT2 shows greater APD and restitution slope dispersion between the base and apex than LQT1. Panel B shows APD dispersion dynamics from the basic long-long CL (grey) followed by a short CL (magenta). The short CL shortens APD in both apex and base, resulting in smaller APD dispersion. Therefore, it creates a uniform DI distribution for the following beat. When CL increases again, a uniform DI distribution (purple) creates a larger APD gradient between apex and base (purple bar in the Y axis) due to steep APD restitution in the base. In addition, the heterogeneous short-term memory effect modulates APD dispersion through further shortening of APD at the base (blue arrows in Fig. 7D,E). Since APD dispersion is reduced by |Δα_2_CL_n−2_|, APD dispersion is greater under short-long CL than long-short CL in LQT2 (see Fig. 7D,E).

The magnitude of α_2_ is about 13% of α_1_ and when large CLs variation occurs such as characteristic short-long RR intervals found during pVT initiation in LQT2 rabbits, its contribution can be estimated to be 28 ms (α_2_ × 700 ms CL = 0.04 × 700 ms), which is a significant impact on APDs in rabbits. If LQT1 and LQT2 are compared, the estimated APD changes by α_2_ by ΔCL = 700 ms will be −7 ms in LQT1 vs. 28 ms in LQT2, showing a greater effect of short-term memory on APDs in LQT2 rabbits.

The heterogeneous short-term memory effect (largest at the base, where APD is largest) can reduce APD dispersion by $|\Delta {\alpha }_{2}C{L}_{n-2}|$. Therefore, when CL_n−2_ is larger (long-long CL case), APD dispersion can be reduced by short-term memory effect and conversely, short CL_n-2_ eliminates the dampening effect by short-term memory to increase APD dispersion. These results indicate that APD dispersion is enhanced under short-long alternating cycles in LQT2 because of small short-term cardiac memory effect caused by short ${{\rm{CL}}}_{n-2}$.

APD dispersion is determined by intrinsic heterogeneity of ion channel expressions. it is well documented that several ionic currents are heterogeneous in the heart, – apico-basal heterogeneity of I_Ks,_ I_to_ and I_Kr_^45,46^, transmural heterogeneity of I_Ks_^47,48^ and I_to_^49^, and RV-LV heterogeneity of I_to_^50^. These ion channel expression patterns can greatly influence short-term cardiac memory and APD dispersion.

The larger short-term cardiac memory effect in LQT2 compared to LQT1 can be linked to the role of I_Ks_ as a dominant repolarizing current when I_Kr_ is absent. Due to its slow activation and inactivation kinetics, I_Ks_ has been linked to short-term cardiac memory^51–53^ and can dynamically create APD dispersion^54^. This is in agreement with the negligible α_2_ in LQT1, where I_Ks_ is lacking. Interestingly, the short-term memory effect is stronger in LMC than LQT2, potentially due to the small reduction of I_Ks_ (~20%) in our transgenic animal model of LQT2^23^. The correlation analysis between the first and the second coefficients also shows a similar tendency, i.e., largest in LMC (−0.41), followed by LQT2 (−0.26), and negligible in LQT1 (−0.07). Under short CL, I_Ks_ (a major determinant of short-term cardiac memory) accumulates due to incomplete deactivation, causing shortening of the APD. Following the long CL, I_Ks_ channels are deactivated, resulting in APD prolongation. Since the expression of I_Ks_ is reported to be heterogeneous^46^, I_ks_ heterogeneity may contribute to enhanced APD dispersion under short-long CL.

Another potential source of short-term cardiac memory is I_to_. The heterogeneous expression of I_to_ has been well documented and influence APD dispersion transmurally, RV vs. LV, and apex-base direction. Due to its relatively rapid turnover of expression, it has been linked to pacing-induced T-wave memory^55,56^. I_to_ has been also implicated in the genesis of APD alternans^57^ and EAD-induced complex APD instability through its modulation of short-term cardiac memory^58^. In our current stimulation protocol with short-long CLs, the short CL_n−2_ can accelerate inactivation of I_to_ and the long CL_n−1_ can provide the sufficient time to recover from the inactivation, which exposes the largest impact of I_to_ heterogeneity on APD and may increase APD dispersion. However, the interpretation should be cautious because I_to_ can influence APD indirectly by modulating other ionic currents and its consequence is difficult to predict. Further studies are needed to delineate its exact roles in cardiac short-term memory and APD dispersion in long QT syndrome.

## Conclusions

In the present study, we demonstrate that APD in LQT2 rabbits is highly dynamic and depends on preceding CL changes. Combined with heterogeneous APD restitution, this property of CL-dependent APD adaptation, known as short-term cardiac memory, promotes greater APD dispersion in LQT2 compared to LQT1 and LMC rabbits. Our results suggest that rate-dependent APD dispersion dynamics plays an important role in determining the genotype-specific initiation of malignant arrhythmias in LQTS.

### Study limitations

Our study was limited to APD dispersion from the anterior surface and transmural mapping of LV without sympathetic stimulation in a narrow short diastolic interval range (<50 ms). This range was chosen to finish pacing protocol without conduction block and tissue-scale PVCs^9,10^ (1/12 random stimulation caused ventricular tachycardia in n = 1/6 LQT2 hearts) and to have relatively linear restitution curve for multivariate analysis. LQT1 hearts demonstrated frequent EADs and pVT induction under isoproterenol, and LQT2 hearts showed time-dependent adaptation of APD under isoproterenol^59^, which makes it difficult to investigate APD dispersion dynamics under isoproterenol with the random CL stimulation protocol. Previous studies^60,61^ indicated that sympathetic nerve stimulation increases APD dispersion due to heterogeneous distribution of sympathetic nerve endings, which cannot be reproduced pharmacologically with isoproterenol. Our study focused on APD dispersion dynamics in a short diastolic interval range (<50 ms) to apply multivariate analysis; however, the relationship between APD dispersion and arrhythmogenesis including reentry or EAD formation remains unclear. Further studies are required to understand the role of APD dispersion in arrhythmogenesis.

## Material and Methods

### Heart preparations

Littermate control (LMC), LQT1, and LQT2 rabbits of both sexes, averaging 16.5 months old/4.2 kg body weight/9.14 g heart weight, were euthanized with buprenorphene (0.03 mg/kg IM), acepromazine (0.5 mg.kg^−1^ IM), xylazene (15 mg.kg^−1^ IM), ketamine (60 mg.kg^−1^ IM), pentothal (35 mg.kg^−1^ IV), and heparin (200 U.kg^−1^). This investigation conformed to the current Guide for Care and Use of Laboratory Animals published by the National Institutes of Health (NIH Publication No. 85-23, revised 1996) and approved by the Lifespan Animal Welfare Committee at Rhode Island Hospital. Hearts were excised and retrogradely perfused through the aorta in a Langendorff perfusion system (Radnoti Glass Technology, Monrovia) with (in mmol.L^−1^) 130 NaCl, 24 NaHCO_3_, 1.0 MgCl_2_, 4.0 KCl, 1.2 NaH_2_PO_4_, 5 Dextrose, 25 Mannitol, 1.25 CaCl_2_, at pH 7.4, and gassed with 95% O_2_ and 5% CO_2_. In total, 23 rabbits were studied: LMC (n = 9), LQT1 (n = 5) and LQT2 (n = 9). Blebbistatin (5 μmol.L^−1^) was perfused to reduce movement artifact^62^.

### Optical mapping

**Figure 8:**
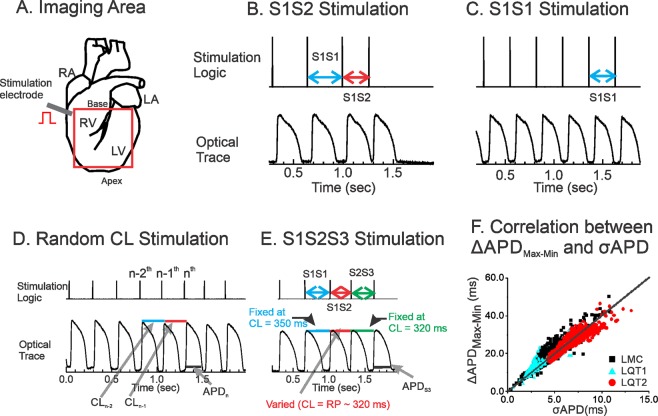
Experimental method. (**A**) Schematics of imaging area and stimulation site. (**B**) S1S2 protocol. (**C**) Ramp pacing. (**D**) Random interval stimulation. CL_n − 1_*;* Activation Interval between n^th^ and n−1^th^ beats. (**E**) S1S2S3 protocol. S1S2 was progressively shortened from 320 ms to the refractory period, while S2S3 was fixed at CL = 320 ms. (**F**) Correlation between σ_APD_ and ΔAPD_max-min_ from LMC (black), LQT1 (blue), LQT2 rabbit hearts (red). ΔAPD_max-min_ and σ_APD_ were calculated from n = 5 hearts per group, about 315 beats per heart during random stimulation. σ_APD,_ equivalent to 0.25 times of ΔAPD_max-min_ (R^2^ = 0.99), is used to estimate APD dispersion to normalized recording region because of robustness to variation in pixel numbers after removing outliers and low-amplitude signals.

The optical apparatus has been previously described^63^. Fluorescence images from the anterior surface and LV free wall of the heart were captured using a CMOS camera (100 × 100 pixels, Ultima-L, SciMedia, Japan), and the field of view was set to 2.0 × 2.0 cm^2^ (spatial resolution of 200 × 200 μm^2^, Fig. 8A). The sampling rate was set to 1,000 frames.s^−1^, and data were analyzed with a custom-built software program developed in Interactive Data Language (Exelis, Inc., Boulder). Hearts were stained with a voltage-sensitive dye, di-4-ANEPPS (Invitrogen, Carlsbad), using 25 μL of stock solution (1 mg.ml^−1^ of dimethyl sulfoxide, DMSO) delivered through a bubble trap, above the aortic cannula. ECG and perfusion pressure were continuously monitored (PowerLab, ADInstruments, Colorado Springs).

#### Stimulation protocol

S1S2 pacing protocol: A conventional S1S2 pacing protocol was applied to measure refractoriness and APD restitution. Typically, 20 beats of constant CL (S1S1 = 350 ms) were applied, followed by a shorter (S1S2) interval. The S1S2 interval was decreased from 300 ms in 10-ms increments until it missed capture (Fig. 8B).

Ramp pacing protocol: After S1S2 and random CL pacing, the hearts were given 10 min to recover prior to ramp pacing (Fig. 8C). Hearts were paced at CLs that were successively decreased by 10 ms until loss of 1:1 capture or induction of ventricular fibrillation or ventricular tachycardia^23,64,65^.

Random CL pacing protocol: The pacing CLs were computer generated using a uniform random number generator, which varied within a 50-ms interval extending from the tissue refractory period measured from S1S2 protocol as described earlier^19^. The 50 ms range of DI was chosen because the dynamic APD restitution curve in this range can be assumed to be linear [^19^ and see Fig. 8D]. In addition, this range of short DI did not allow additional triggered activity such as early afterdepolarizations, so that multiple linear regression analysis can be applied (Fig. 8D).

S1S2S3 pacing protocol: To quantify the short-term memory effect on APD and APD dispersion, the S1S2 stimulation protocol was extended to include S2S3 stimulation, in which the S2S3 interval was fixed at 320 ms but the S1S2 was varied. Similar to S1S2 pacing, 20 beats of constant CL (S1S1 = 350 ms) were applied, followed by a variable S1S2 (from 300 ms to the refractory period with a 10 ms increment) and a fixed S2S3 at 300 or 320 ms, and the APD and APD dispersion of *beat S3* were plotted as a function of *S1S2*. Since S2S3 is fixed, this pacing protocol measures the effect of S1S2 on APD_n_ and its dispersion exclusively without the influence from CL_n−1_ (Fig. 8E).

### Data analysis

The activation and repolarization time points at each site were determined from fluorescence (F) signals by calculating (dF/dt)_max_ and (d^2^F/dt^2^)_max_. Data were filtered using a spatial Gaussian filter (3 × 3 pixel), and first/second derivatives were calculated using a temporal polynomial filter (3^rd^ order, 13 points). Pixels with low signal-to-noise ratio determined by (dF/dt)_max_ (lower than 3 × σ of baseline) and outliers of pixels determined by Grubbs’ test were removed from analysis.

APD dispersion was defined as standard deviation of APD (σ_APD_), because the standard deviation was robust to variation in pixel numbers after removing outliers and low-amplitude signals. σ_APD_ and the ΔAPD_max-min_, which was defined as differences between 99% quartile (APD_max_) and 1% quartile (APD_min_) of spatial APD distributions, have positive correlation with 4.01 coefficient value as shown in Fig. 8F, and σ_APD_ is equivalent to 1/4^th^ of ΔAPD_max-min_.

#### Multivariate analysis of APD and previous CLs

Multivariate analysis of APD from random CL stimulation was carried out as previously described^19^ to quantify the short-term cardiac memory effect. Briefly, CLs were calculated by subtracting the previous activation time from the next activation time. APDs were measured from individual pixels and as a result, series of CLs (CL_1_, CL_2_, …, CL_n_) and APDs (APD_1_, APD_2_, …, APD_n_) were calculated (see Fig. 8D). Then each APD was curve-fitted to the previous CLs using a linear regression model^19^. For example, each APD was fitted to its previous *k*^*th*^ CLs as:

1 $$AP{D}_{n}=C+{\alpha }_{1}C{L}_{n-1}+{\alpha }_{2}C{L}_{n-2}+\,\cdots +\,{\alpha }_{k}C{L}_{n-k}\,$$

A minimum of 80 APs were analyzed for each multivariate linear regression model. At least three independent scans were analyzed from the same heart to verify reproducibility of the curve fitting.

## Supplementary information

Supplementary Information

Mechanisms of short-long CL enhancing APD dispersion in LQT2
