# Supplementary material for: Short-Long Heart Rate Variation Increases Dispersion of Action Potential Duration in Long QT Type 2 Transgenic Rabbit Model
Source: Sci Rep. 2019 Oct 16;9:14849. doi: 10.1038/s41598-019-51230-9 (PMC6795902; doi:10.1038/s41598-019-51230-9)
Supplement: Supplementary file 1 — Supplementary Information [file 41598_2019_51230_MOESM1_ESM.docx]

**Supplementary Information**

**Title**

**Short-Long Heart Rate Variation Increases Dispersion of Action Potential Duration in Long QT Type 2 Transgenic Rabbit Model**

**Authors:**

Tae Yun Kim^1^, Paul Jeng^1^, JungMin Hwang^2^, Zachary Pfeiffer^1^, Divyang Patel^3^, Leroy L Cooper^4^, Konstantinos Kossidas^1^, Jason Centracchio^1^, Xuwen Peng^5^, Gideon Koren^1^, Zhilin Qu^6^, and Bum-Rak Choi^1^

^1^Cardiovascular Research Center, Division of Cardiology, Rhode Island Hospital, Warren Alpert Medical School of Brown University, Providence, RI

^2^College of Pharmacy, University of Rhode Island, Kingston, RI

^3^Department of Cardiovascular Medicine, Heart and Vascular Institute, Cleveland Clinic Foundation, Cleveland, Ohio

^4^Biology Department, Vassar College, Poughkeepsie, NY

^5^Department of Comparative Medicine, Pennsylvania State University College of Medicine,

Hershey, PA.

^6^Department of Medicine (Cardiology), David Geffen School of Medicine, University of California, Los Angeles, CA

To whom all correspondence should be addressed: [Bum-Rak_Choi@brown.edu](mailto:Bum-Rak_Choi@brown.edu)


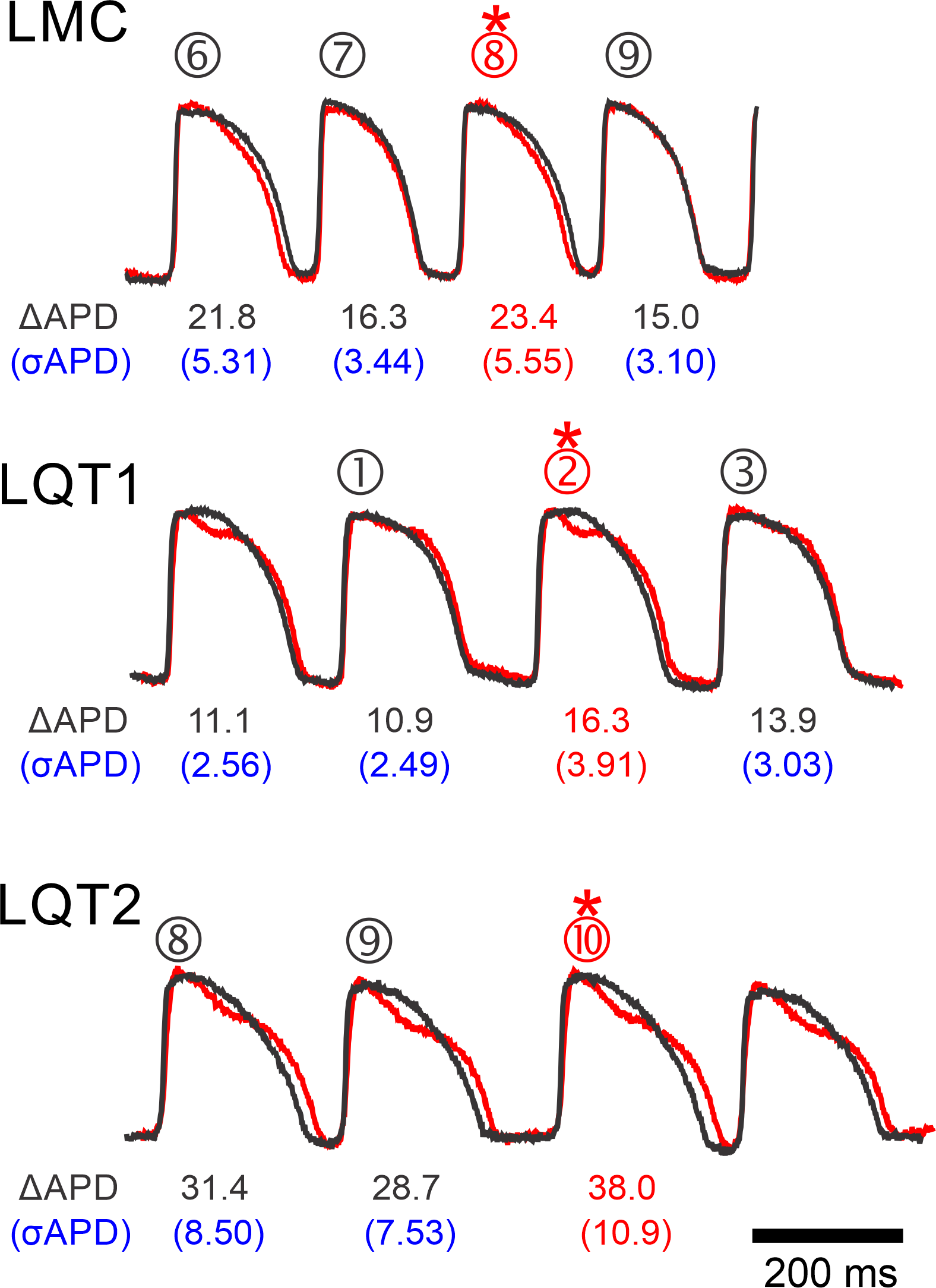


**Supplementary Figure 1**. Detailed action potential traces from LMC, LQT1, and LQT2 rabbit hearts in Figure 1-3 during random pacing. Two traces are chosen from the base and apex of the anterior surface to show differences in the repolarization phase. The largest repolarization gradient was observed from LQT2 during short-long pacing CLs.

**Supplementary Video File 1.** The step-by-step illustration of Figure 7 that explains the mechanisms of short-long CL enhancing APD dispersion in LQT2. Consider a long-long…long-short-long stimulation sequence, and at the steady-state long CL pacing, the tissue resolves steady APD and DI distribution patterns (grey bars). In the short CL beat, the heterogeneous APD distribution of the previous beat results in a larger DI dispersion, which gives rise to a smaller APD dispersion (magenta bars). Due to the smaller difference in APD, the DI difference preceding the next long CL beat is reduced (purple bar), which results in a APD difference larger than that in the long-long pacing cycles (comparing the vertical grey and magenta bars). This shows that a long-short-long cycle pacing sequence amplifies APD dispersion in the presence of heterogeneous APD restitution slopes, which is what occurs in the LQT2 hearts.

In addition to heterogeneous APD restitution, heterogeneous memory effect can further increase APD dispersion. From results of multivariable regression in random pacing (${APD}_{n}=C+ \alpha_{1}{CL}_{n-1}+ \alpha_{2}{CL}_{n-2}$) , the expanded APD restitution in the base and apex can be approximated to

$${APD}_{n}^{Base}=C_{Base}^{'}+ \alpha_{1}^{Base}{DI}_{n-1}^{Base}+ \alpha_{2}^{Base}{CL}_{n-2}^{Base}$$

,

$${APD}_{n}^{Apex}=C_{Apex}^{'}+ \alpha_{1}^{Apex}{DI}_{n-1}^{Apex}+ \alpha_{2}^{Apex}{CL}_{n-2}^{Apex}$$

and, the APD difference is,

$${\Delta APD}_{n}= C^{'}+\alpha_{1}^{Base}{DI}_{n-1}^{Base}- \alpha_{1}^{Apex}{DI}_{n-1}^{Apex}+(\alpha_{2}^{Base}- \alpha_{2}^{Apex}){CL}_{n-2}$$

where, $\alpha_{1}^{Base}$and $\alpha_{1}^{Apex}$ are the slopes of the S1S2 APD restitution, $\alpha_{2}^{Base}$ and $\alpha_{2}^{Apex}$ are second previous beat interval dependent coefficient of the base and apex. Collectively, $\alpha_{2}^{Base}{CL}_{n-2}$ and $\alpha_{2}^{Apex}{CL}_{n-2}$ quantify the memory effect found in the base and apex regions. APD dispersion induced by only APD restitution heterogeneity is shown in Figure 7C (blue bar). Regarding the cardiac memory effect shown in Figure 5C, since $\alpha_{2}^{Base}$ and $\alpha_{2}^{Apex}$ are negative, the cardiac memory effect shortens APD, corresponding to $\alpha_{2}^{Base}{CL}_{n-2}$ and $\alpha_{2}^{Apex}{CL}_{n-2}$ (blue arrows in Figure 7D, E). Also, because $\alpha_{2}^{Base}$ and $\alpha_{2}^{Apex}$ have negative correlation with each APD restitution slope ($\alpha_{1}^{Base}$and$\alpha_{1}^{Apex}$) and $\alpha_{1}^{Base}<\alpha_{1}^{Apex}$ as shown in Figure 5B, the amount of CL_n-2_ coefficient in the base was smaller than in the apex ($\left|\alpha_{2}^{Base}\right|<\left|\alpha_{2}^{Apex}\right|$). Therefore, the memory effect-dependent APD difference between the base and apex ($(\alpha_{2}^{Base}- \alpha_{2}^{Apex}{)CL}_{n-2}$) is always positive. Therefore, the heterogeneous memory effect can further increase APD dispersion as depicted in Figure 7D, E (magenta bar).
